# Rapid and Sensitive Quantification of the Pesticide Lindane by Polymer Modified Electrochemical Sensor

**DOI:** 10.3390/s21020393

**Published:** 2021-01-08

**Authors:** Jafar Safaa Noori, John Mortensen, Alemnew Geto

**Affiliations:** 1 IPM—Intelligent Pollutant Monitoring ApS, 2690 Karlslunde, Denmark; alg@intpm.dk; 2 Department of Science and Environment, Roskilde University, 4000 Roskilde, Denmark; john@ruc.dk

**Keywords:** pesticide, lindane, polymer, electrochemical, sensor, water, contamination

## Abstract

Lindane is documented by the Environmental Protection Agency (EPA) as one of the most toxic registered pesticides. Conventional detection of lindane in the environment requires manual field sampling and complex, time-consuming analytical sample handling relying on skilled labor. In this study, an electrochemical sensing system based on a modified electrode is reported. The system is capable of detecting lindane in aqueous medium in only 20 s. The surface of a conventional carbon electrode is modified with a film of conductive polymer that enables detection of lindane down to 30 nanomolar. The electrode modification procedure is simple and results in a robust sensor that can withstand intensive use. The sensitivity of the sensor is 7.18 µA/µM and the performance was demonstrated in the determination of lindane in spiked ground water. This suggests that the sensor is potentially capable of providing useful readings for decision makers. The rapid and sensitive quantification of lindane in aqueous medium is one step forward to new opportunities for direct, autonomous control of the pesticide level in the environment.

## 1. Introduction

Lindane (1α, 2α, 3β, 4α, 5α, 6β-hexachlorocyclohexane) is a polychlorinated insecticide used in agriculture to protect fruits, vegetables and crops from insects, eggs and larvae [1]. Lindane has the potential to significantly alter ecosystems, pose toxicity to animals and humans and be accumulated in the food chain. The heavy use of lindane in agriculture has led to contamination of the environment [2]. Exposure to lindane may occur from eating contaminated food or by drinking contaminated water [3]. Intake of lindane is associated with neurotoxic effects and may cause anemia and significant damage to the liver, and the immune system [4].

Currently, various analytical methods are available for the detection and quantification of lindane in the environment [5]. Common for most is that they involve collection of samples, retrieval of the samples to a central laboratory and labor-intensive sample clean-up and pretreatment steps followed by quantification [6]. In addition to the lengthy and uneconomical steps involved, degradation of lindane may occur during transportation of the samples from the field causing inaccurate analytical results [7]. Thus, it is desirable to develop techniques that enable rapid in situ measurements in the environment. Electrochemical sensors present a promising alternative to conventional determination of lindane in the environment due to its rapid and sensitive measurement protocol. Furthermore, recent advances in electronics has enabled electrochemical sensors to have the potential to be employed for onsite detection [1,8,9].

The first step towards onsite electrochemical detection of lindane in the environment is to develop selective detection of the compound. However, only few reports are available in the literature regarding the electrochemistry of lindane in conventional and modified electrodes. Earlier reports focused on the reduction of the compound on mercury based electrodes [10] which is currently a concern of health and environmental pollution itself. Other electrochemical studies of lindane on modified and non-modified electrodes were available either in a completely organic media [11,12,13,14] or aqueous-organic mixtures [15,16,17,18] making them less attractive for direct environmental application. Moreover, the peak potential for the reduction of lindane was reported to occur at −1.45 V [18], −1.5 V [15,17,19] −1.94 V (vs. Ag/AgCl) [11], or −1.40 and −2.10 V [12,14], which is highly negative for selective determination due to interference problems from co-existing compounds. A study [16], on the reduction of lindane at silver cathodes in organic and aqueous-organic media showed a shift of the reduction peak to less negative potential with the addition of water from −1.40 V in 100% acetonitrile (ACN) to −0.89 V in 50:50 ACN-H_2_O. This shows the significant influence of the medium in the reduction potential of lindane shifting positively with increasing the water proportion. Therefore, the objective of this study is to develop an electrochemical method for the determination of lindane in a completely aqueous medium using a polymer modified carbon electrode.

The polymer film used in this study was electrochemically synthesized from 4-amino-3-hydroxynaphtahele sulfonic acid (4A3HNSA) in 0.1 HNO_3_. The polymer deposition process and the property of the resulting film was characterized using cyclic voltammetry(CV), scanning electron microscopy(SEM) and electrochemical impedance spectroscopy(EIS) [20] and it was found to result in remarkably robust electrodes. The polymer film modified glassy carbon electrode has been reported previously for the determination of various bioactive molecules [21,22,23]. However, all previous uses of the electrode were limited on the oxidation of the compounds and the current report is the first attempt to apply it in the reductive detection.

## 2. Experimental

### 2.1. Reagents and Solutions

Chemicals and reagents used in this study were all analytical grade and used as received without any pretreatment. Aqueous solutions were prepared in Milli-Q water (resistivity ≥ 18 MΩ cm). Lindane (1α, 2α, 3β, 4α, 5α, 6β-hexachlorocyclohexane), 4-amino-3-hydroxynaphthalene sulfonic acid, chlorobenzene, 4-chlorobenzaldehyde, 1,3,5-trichlorobenze and *N*,*N*-Dimethylformamide (DMF) were all supplied by Sigma-Aldrich. KCl was purchased from Frederiksen Scientific A/S. 10 mM stock solution was prepared by dissolving lindane in *N*,*N*-Dimethylformamide (DMF) and kept in brown bottle in the fridge. All working solutions were prepared by diluting appropriate volume of the stock solution in Milli-Q water containing supporting electrolyte.

Ground water sample was analyzed by spiking different concentrations of lindane. The water sample was collected from Gevninge waterworks, Southern Copenhagen directly after pumping it out from the well. Until analysis, the sample was stored in a tight container to avoid oxygen contact and kept in the dark at 4 °C.

### 2.2. Experimental Setup

**Figure 1:**
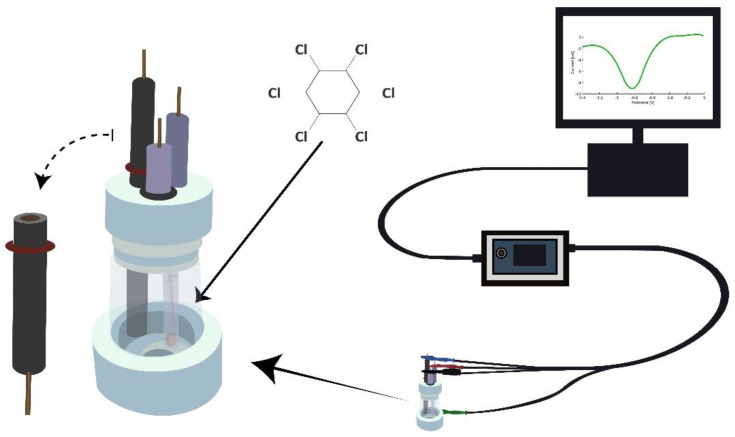
Electrochemical setup used in the study consisting of electrochemical cell, a potentiostat connected to a computer system to run the measurement and record data.

All electrochemical measurements were conducted in a conventional three electrode setup as seen in Figure 1. The electrochemical cell consists of bare or polymer modified glassy carbon electrode (3 mm diameter) as a working electrode, Pt wire as counter electrode and Ag/AgCl reference electrode. The electrodes were connected to a potentiostat (EmStat 3+, PalmSense, The Netherlands) and a computer system from which the applied settings were controlled. All data analyses were conducted with PSTrace 5.5 (PalmSense, Houten, The Netherlands).

### 2.3. Preparation of the Working Electrode

Prior to use, glassy carbon electrode was polished with 0.3 and 0.05 µm alumina slurry on a polishing pad and rinsed thoroughly with Milli-Q water. The clean electrode was placed in 2 mM monomer solution which was prepared by dissolving 4-amino-3-hydroxynaphthalene sulfonic acid in 0.1 M HNO_3_. Electrochemical polymerization was conducted by scanning the potential between −0.8 V and +2.0 V using cyclic voltammetry for 15 cycles at 0.1 V s^−1^, a thin film of the conducting polymer is formed at the electrode surface. Cyclic voltammetry scans between −0.8 V and +0.8 V in 0.5 M H_2_SO_4_ was conducted to stabilize the film and to remove any non-adsorbed items from the electrode surface. Further details about the film formation and characterization can be found in a previous report [20].

### 2.4. Electrochemical Measurements

Cyclic voltammetry (CV) and square wave voltammetry (SWV) were used to study the electrochemical behavior of lindane. In the studies, 10 mL of 0.2 M KCl solution was placed in the electrochemical cell to record the background response and appropriate volume of 10 mM lindane in DMF was added to it and mixed thoroughly to record voltammograms of the compound. SWV was used to study the electrochemical behavior in the absence and presence of 100 µM lindane at bare and polymer modified glassy carbon electrode. The effect of scan rate was studied by running CV measurements in 100 µM lindane solution between 40 and 400 mV s^−1^ in the potential window from 0.0 to −2.0 V. The interference study was also investigated using SWV in the absence and presence of equal amount of interfering compound in 1 µM lindane solution.

In spiked sample analysis, 10 mL of 20:80 ground water: 0.2 M KCl was placed and background signal recorded. Then, this mixture was spiked with three concentration of lindane systematically chosen at different levels of the standard calibration curve to make 0.1, 0.5 and 1 µM and the response was obtained. The current response from the background corrected voltammogram was plotted against lindane concentration.

## 3. Results and Discussion

### 3.1. Electrochemical Behavior of Lindane

**Figure 2:**
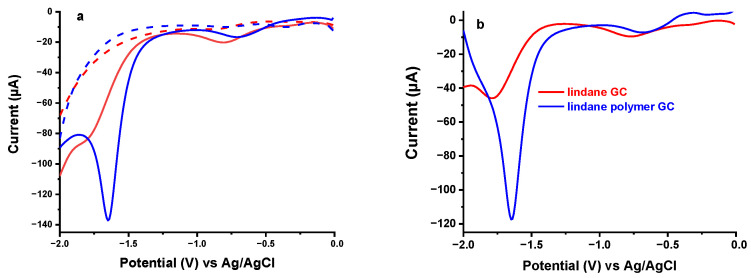
Square wave voltammogram in the absence (broken) and presence (solid) of lindane in 0.2 M KCl solution at bare (red) and polymer modified (blue) glassy carbon electrode (**a**) and background corrected response at bare (red) and polymer modified (blue) electrode (**b**). SWV conditions: E_step_ = 0.01 V; Amplitude = 0.1 V; Frequency = 15.0 Hz.

**Figure 3:**
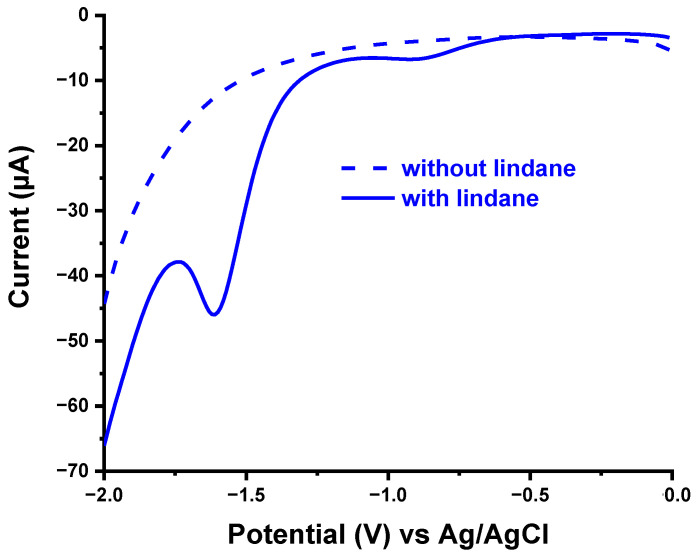
Square wave voltammogram in the absence (broken) and presence (solid) of lindane in nitrogen saturated 0.2 M KCl solution at glassy carbon electrode. SWV conditions: E_step_ = 0.01 V; Amplitude = 0.1 V; Frequency = 15.0 Hz.

The electrochemical profile of lindane was studied by square wave voltammetry at a bare and polymer modified glassy carbon electrode in 0.2 M KCl solution (Figure 2). At both electrodes, the voltammograms showed two distinct peaks in the region around −1.60 V and −0.80 V in the presence of 100 µM lindane (solid lines) while no significant peaks were observed in the absence of lindane (broken lines). The peak around −1.6 V is the significant signal which is also consistent with previous reports [11,12,14,15,17,18] regarding the reduction of lindane at various electrodes. This peak is attributed to the transformation of lindane to benzene through electrochemical reduction involving 6-electrons [24]. The second weak peak observed around −0.8 V is not reported in previous studies except in one stating the oxygen mediated reduction of lindane facilitated by the reaction of superoxide from oxygen reduction with lindane [11]. However, preliminary studies in nitrogen saturated medium in the absence and presence of lindane (Figure 3) revealed a consistent reduction peak confirming the absence of oxygen involvement in the process. Rather, the complete reduction of lindane to benzene might be involving a series of intermediate reduction steps by breaking a carbon-chlorine bond to form less chlorinated compounds. The reduction of lindane at lower negative potential is also in agreement with a study by Peverly et al., who reported a shift in the reduction potential to more positive values with an increase in the amount of water during aqueous-organic mixture media at a silver electrode [16]. The effective proton donor property of water is thought to promote the conversion of lindane to benzene.

Comparing the bare and the polymer modified electrodes, the peak current obtained at the modified electrode is five-times higher than the current recorded at the bare electrode at the more negative potential. Whereas, no significant difference in peak response is observed between the electrodes at the less negative region. In addition to this, the reduction peak potentials also shifted positively from −1.79 V to −1.64 V at high negative region and from −0.81 V to −0.69 V at lower negative region using the bare and modified electrodes, respectively. This shows the catalytic property of the polymer film on the electrochemical reduction of lindane. The better performance of the polymer modified electrode might be attributed to the presence of -OH, -SO_3_H and -NH_2_ functional groups in the naphthalene-based polymer film that can contribute to catalytic reduction of lindane. In addition, the naphthalene skeleton of the polymer by itself can better interact with organic and non-polar molecules and could play a role in the better performance. Therefore, the polymer modified electrode is selected for subsequent experiments.

### 3.2. Effect of Potential Scan Rate and Buffer pH

**Figure 4:**
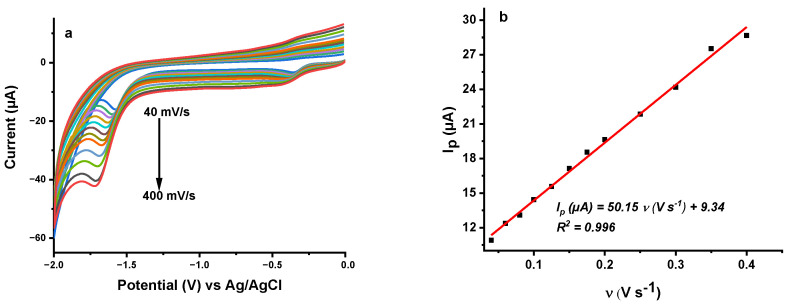

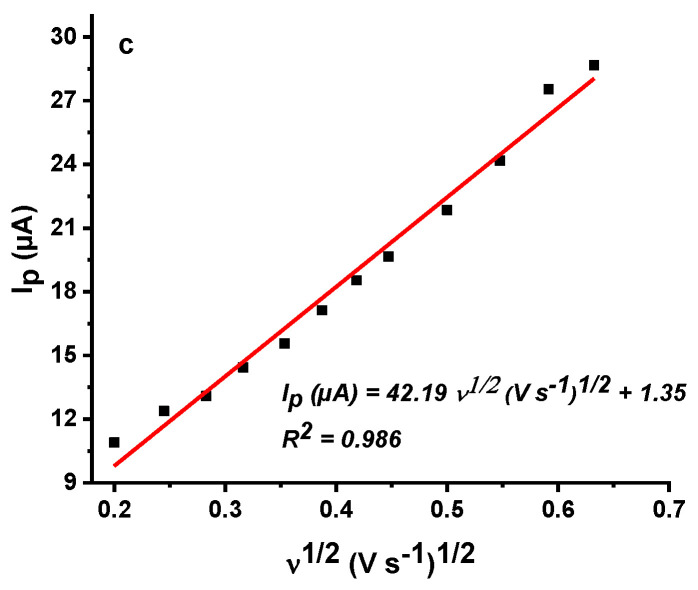
(**a**) cyclic voltammograms of 100 µM lindane using different scan rates in 0.2 M KCl solution and reduction peak currents extracted from the voltammograms plotted against the scan rates (**b**) and against the square root of scan rates (**c**).

Cyclic voltammograms of 100 µM lindane were recorded in 0.2 M KCl solution with different scan rates to investigate the reduction kinetics at the polymer modified electrode (Figure 4). The study was made between 40 and 400 mV s^−1^ and the peak current at ~−1.6 V was plotted against the scan rate and square root of scan rate, respectively, which the plots yield the linear regression equations: I_p_ (µA) = 50.14 ν (V s^−1^) + 9.34 (R^2^ = 0.996) and I_p_ (µA) = 42.19 ν^1/2^ (V^1/2^ s^−1/2^) + 1.35(R^2^ = 0.986). These results depict that the kinetics of lindane reduction at the modified electrode has contributions from both adsorption and diffusion processes [25]. However, the plot of the log peak current (µA) against log scan rate (V s^−1^) also followed a linear relation according to the equation: log I_p_ (µA) = 0.433 log ν (V s^−1^) + 1.608 (R^2^ = 0.978), with a slope of 0.433 close to 0.5 a theoretical value for a purely diffusion-controlled process, indicating the dominant effect of diffusion in the reduction process [26]. Additionally, a shift in the reduction peak to more negative potential is observed from the plot of E_p_ vs log scan rate following an equation: E_p_ (V) = 0.140 log ν (V s^−1^) + 1.772 (R^2^ = 0.992) which confirms the complete irreversibility of the reduction process.

The effect of solution pH on the reduction of lindane at the polymer modified electrode was investigated in 0.1 M phosphate buffer solution of pH 4 to 9. Square wave voltammetry was chosen over cyclic voltammetry in this study for better peak resolution and measurements were recorded in 100 µM lindane in different pH conditions. The results did not show a shift in peak potentials with change in pH which is in agreement with previous reports [10]. However, increasing in buffer pH led to a decrease in peak currents which might be due to the conductivity loss or film degradation in higher pH solutions as previously noted in the polymer film [20]. For practical convenience, subsequent experiments were performed in 0.2 M KCl as supporting electrolyte.

### 3.3. Rapid Quantification of Lindane in Aqueous Solution

The quantification of lindane using the modified electrode was performed using square wave voltammetry. The reduction potential at ~−0.8 V was targeted as the characteristic peak for lindane quantification as it appears at relatively lower negative potential than the strong peak at ~−1.6 V. A lower negative potential is preferable to avoid interference from co-existing compounds. Furthermore, preliminary tests show that the peak at ~−1.6 V appears only at higher lindane concentrations which makes the second peak the dominant signal in low concentrations and practically relevant for sensitive determination of lindane.

**Figure 5:**
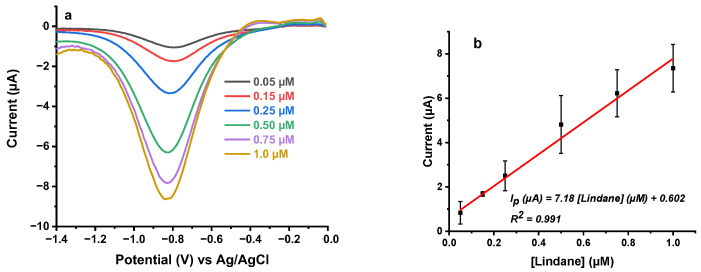
Calibration curve of lindane in 0.2 M KCl using (**a**) square wave voltammetry and (**b**) plot of extracted peak current against lindane concentration. SWV conditions: E_step_ = 0.01 V; Amplitude = 0.1 V; Frequency = 15.0 Hz.

Square wave voltammograms between 0.0 to −1.4 V were recorded to quantify lindane in the concentration range from 0.05 to 1.0 µM in 0.2 M KCl solution (Figure 5a). The background measurement was recorded in 0.2 M KCl and then a series of lindane standards were spiked to the solution, stirred thoroughly and measurements were performed. The background corrected voltammograms Figure 5a and the mean peak currents extracted from three independent calibration measurements together with standard deviation used as error bars were plotted (Figure 5b). Figure 5b shows that the peak current is linearly proportional to the lindane concentration with the sensitivity of 7.18 µA/µM. The linear regression equation of the plot was: I_p_ (µA) = 7.18 [lindane] (µM) + 0.602 (R^2^ = 0.991).

The theoretical detection and quantitation limits were calculated following the International Conference on Harmonisation (ICH) guideline [27] by 3.3 × *δ*/*s* and 10 × *δ*/*s*, respectively; where ‘*δ*’ is the standard deviation of the y-intercept of the regression line and ‘*s*’ is the slope of calibration curve. Thus, the calculated detection limit (LoD) was found to be 30 nM while the quantitation limit (LoQ) was 98 nM. The regulatory limit of pesticides in drinking water is 0.1 µg/L which corresponds to 0.34 nM [28]. This limit of detection is lower than electrochemical methods reported for lindane determination at cellulose acetate/GC [15], MnO_2_/GC [18], NiCo_2_O_4_/GC [17] and comparable with vitreous carbon [14], Nylon 6.6/MWCNT/Fe_3_O_4_ [29] and CuO–MnO_2_/GC [19]. Moreover, this study is the first attempt to determine lindane in a completely aqueous medium. Summary and comparison of electrochemical methods and their analytical performance in the determination of lindane is shown in Table 1.

### 3.4. Interference Study

For the interference study, chlorobenzene, 4-chlorobenzaldehyde and 1,3,5-trichlorobenzene were selected for their structural similarity to the target molecule, lindane. Square wave voltammograms were recorded using the modified electrode in 1 µM lindane solution in the presence and absence of equal concentration of interfering compounds. The effect of interference was estimated from the relative error by calculating the percentage ratio of the peak current difference in the presence to peak current in the absence of interferent. The relative error obtained in the presence equal molar ratio of chlorobenzene, 4-chlorobenzaldehyde and 1,3,5-trichlorobenzene is −0.47%, −1.5% and 1.8%, respectively, indicating absence of any interference effect in the determination of lindane. Therefore, the developed sensor has a potential for the selective determination of lindane in the presence of structurally similar compounds.

### 3.5. Promising Use in the Field

The aim of using electrochemical sensors for lindane detection is the promise of device deployment in the fields to avoid manual sampling and centralized sample analyses. Electrochemistry is an emerging technology that has the possibility to identify and quantify different compounds and measure concentrations lower than regulation guidelines requirements. The recent breakthrough in electronics and communication technologies allow the electrochemical method to be applied as a field measuring technique by having a small portable measuring device. This study shows for the first time the simple detection of lindane by polymer-modified sensors with a detection range relevant for field applications.

With the possibility of deploying electronics directly in the field, measurements can be recorded as frequent as relevant. With a measurement time of only 20 s, this technique outcompetes manual sampling, transportation and lengthy analyses of the pesticide content in laboratories. Real-time, online measurements can allow immediate intervention when a site is proven to be contaminated.

The stability of the deposited polymers on the electrode is an important factor when conducting frequent measurements in the field. The modified electrode was stable for repeated use for more than a week after keeping it in an ambient condition. In a previous report, the electrode showed an excellent shelf life stability by maintaining 97.6% of its initial activity after 25 days of storage in a refrigerator [21].

A real sample from groundwater was spiked with three different concentrations of lindane, 0.1 µM, 0.5 µM and 1µM. The spiked samples were then tested using the modified electrodes resulting in a linear fit of 0.991 and a slope of 0.739 which does not match the slope presented in Figure 5b. This difference in the slope could be due to the matrix effect of the sample or the change in the ionic strength of the medium when mixed with the ground water [8,30]. In addition, the unknown groundwater sample could contain substances that may bind to the polymer and hence cause the shift of the measured signals.

## 4. Conclusions

Pesticides have been a powerful tool to increase crop yield in the agriculture, however, pesticides are contaminating our environment and polluting our drinking water. To warn consumers about contaminated water, the ultimate goal is to conduct frequent control of the water quality directly in the field. Continuous electrochemical detection of pesticides in the field coupled with an alert system that warns the water suppliers when the pesticide level is exceeding the regulation threshold will make it possible to minimize human exposure to critical pesticides as lindane. Future studies should focus on the development of a full detection system that can be deployed in the field and provide frequent readings on the contaminant level.

## Figures and Tables

**Table 1:** Comparison of different electrochemical methods reported for the determination of lindane.

Electrode	Medium	Method	Linear Range	LoD	E_Peak_ (V)	Reference
**Cellulose Acetate/GC**	60:40 MeOH-H_2_O	DPV	50–180 µM	9.18 µM	−1.5	[15]
**Vitreous carbon**	EtOH	SWV		50 nM	−2.0	[14]
**MnO_2_/GC**	60:40 MeOH-H_2_O	Amperometry	1.1–510 µM	114 nM	−1.45	[18]
**Nylon 6,6/MWCNT/Fe_3_O_4_**	60: 40 MeOH-H_2_O	SWV	9.9 pM–5 µM	32 nM	−0.49	[26]
**NiCo_2_O_4_/GC**	60:40 MeOH-H_2_O	DPV	10–100 µM	5.9 µM	−1.5	[17]
**CuO–MnO_2_/GC**	60:40 MeOH-H_2_O	DPV	1–700 µM	4.8 nM	−1.5	[27]
**Vitreous carbon**	DMF	SWV	40−1000 µM		−1.94	[11]
**Polymer/GC**	H_2_O	SWV	0.05–1 µM	30 nM	−0.82	This work

## References

[1] Boni A.C., Wong A., Dutra R.A.F., Sotomayor M.D.P.T. (2011). Cobalt phthalocyanine as a biomimetic catalyst in the amperometric quantification of dipyrone using FIA. Talanta.

[2] Carvalho F.P. (2017). Pesticides, environment, and food safety. Food Energy Secur..

[3] Dalvie M.A., Cairncross E., Solomon A., London L. (2003). Contamination of rural surface and ground water by endosulfan in farming areas of the Western Cape, South Africa. Environ. Health.

[4] Parmar D., Yadav S., Dayal M., Johri A., Dhawan A., Seth P.K. (2003). Effect of lindane on hepatic and brain cytochrome P450s and influence of P450 modulation in lindane induced neurotoxicity. Food Chem. Toxicol..

[5] Muir D.C., Sverko E. (2006). Analytical methods for PCBs and organochlorine pesticides in environmental monitoring and surveillance: A critical appraisal. Anal. Bioanal. Chem..

[6] Ruzicka J., Hansen E.H. (2008). Retro-review of flow-injection analysis. TrAC Trends Anal. Chem..

[7] Girish K., Mohammad Kunhi A.A. (2013). Microbial degradation of gamma-hexachlorocyclohexane (lindane). Afr. J. Microbiol. Res..

[8] Geto A., Noori J.S., Mortensen J., Svendsen W.E., Dimaki M. (2019). Electrochemical determination of bentazone using simple screen-printed carbon electrodes. Environ. Int..

[9] Noori J.S., Dimaki M., Mortensen J., Svendsen W.E. (2018). Detection of Glyphosate in Drinking Water: A Fast and Direct Detection Method without Sample Pretreatment. Sensors.

[10] Beland F.A., Farwell S.O., Robocker A.E., Geer R.D. (1976). Electrochemical reduction and anaerobic degradation of lindane. J. Agric. Food Chem..

[11] Birkin P.R., Evans A., Milhano C., Montenegro M., Pletcher D. (2004). The Mediated Reduction of Lindane in DMF. Electroanalysis.

[12] Merz J.P., Gamoke B.C., Foley M.P., Raghavachari K., Peters D.G. (2011). Electrochemical reduction of (1R,2r,3S,4R,5r,6S)-hexachlorocyclohexane (Lindane) at carbon cathodes in dimethylformamide. J. Electroanal. Chem..

[13] Matsunaga A., Yasuhara A. (2005). Dechlorination of polychlorinated organic compounds by electrochemical reduction with naphthalene radical anion as mediator. Chemosphere.

[14] Martins P.C., Medeiros M.J., Montenegro M.I. (1999). Electrochemical behaviour of hexachlorocyclo-hexane. Port. Electrochim. Acta.

[15] Kumaravel A., Vincent S., Chandrasekaran M. (2013). Development of an electroanalytical sensor for γ-hexachlorocyclohexane based on a cellulose acetate modified glassy carbon electrode. Anal. Methods.

[16] Peverly A.A., Karty J.A., Peters D.G. (2013). Electrochemical reduction of (1R,2r,3S,4R,5r,6S)-hexachlorocyclohexane (Lindane) at silver cathodes in organic and aqueous–organic media. J. Electroanal. Chem..

[17] Prathap M.A., Srivastava R. (2013). Electrochemical reduction of lindane (γ-HCH) at NiCo_2_O_4_ modified electrode. Electrochim. Acta.

[18] Prathap M.U.A., Sun S., Xu Z.J. (2016). An electrochemical sensor highly selective for lindane determination: A comparative study using three different α-MnO2 nanostructures. RSC Adv..

[19] Prathap M.U.A., Sun S., Wei C., Xu Z.J. (2015). A novel non-enzymatic lindane sensor based on CuO–MnO_2_ hierarchical nano-microstructures for enhanced sensitivity. Chem. Commun..

[20] Geto A., Brett C.M. (2016). Electrochemical synthesis, characterisation and comparative study of new conducting polymers from amino-substituted naphthalene sulfonic acids. J. Solid State Electrochem..

[21] Geto A., Amare M., Tessema M., Admassie S. (2012). Voltammetric Determination of Nicotine at Poly(4-Amino-3-Hydroxynaphthalene Sulfonic Acid)-Modified Glassy Carbon Electrode. Electroanalysis.

[22] Tefera M., Geto A., Tessema M., Admassie S. (2016). Simultaneous determination of caffeine and paracetamol by square wave voltammetry at poly(4-amino-3-hydroxynaphthalene sulfonic acid)-modified glassy carbon electrode. Food Chem..

[23] Geto A., Tessema M., Admassie S. (2014). Determination of histamine in fish muscle at multi-walled carbon nanotubes coated conducting polymer modified glassy carbon electrode. Synth. Met..

[24] Farwell S.O., Beland F.A., Geer R.D. (1975). Interrupted-sweep voltammetry for the identification of polychlorinated biphenyls and naphthalenes. Anal. Chem..

[25] Bard A.J., Faulkner L.R. (2001). Electrochemical Methods, Fundamentals and Applications.

[26] Kalaiyarasi J., Meenakshi S., Gopinath S.C.B., Pandian K. (2017). Mediator-free simultaneous determination of acetaminophen and caffeine using a glassy carbon electrode modified with a nanotubular clay. Microchim. Acta.

[27] Shrivastava A., Gupta V.B. (2011). Methods for the determination of limit of detection and limit of quantitation of the analytical methods. Chron. Young Sci..

[28] Noori J.S., Mortensen J., Geto A. (2020). Recent Development on the Electrochemical Detection of Selected Pesticides: A Focused Review. Sensors.

[29] Fayemi O.E., Adekunle A.S., Ebenso E.E. (2016). A Sensor for the Determination of Lindane Using PANI/Zn, Fe(III) Oxides and Nylon 6,6/MWCNT/Zn, Fe(III) Oxides Nanofibers Modified Glassy Carbon Electrode. J. Nanomater..

[30] Lezi N., Economou A. (2015). Voltammetric Determination of Neonicotinoid Pesticides at Disposable Screen-Printed Sensors Featuring a Sputtered Bismuth Electrode. Electroanalysis.

